# Combined Preimplantation Genetic Testing for Aneuploidy (PGT-A) and Monogenic Disorders (PGT-M) to Prevent Autosomal Dominant Polycystic Kidney Disease After Kidney Transplant

**DOI:** 10.7759/cureus.95728

**Published:** 2025-10-30

**Authors:** Wael Elbanna, Osama Azmy, Manal A Elhinnawi

**Affiliations:** 1 Obstetrics and Gynecology, Hayat Women Care Center, Cairo, EGY; 2 Obstetrics and Gynecology, Egypt Center for Research and Regenerative Medicine (ECRRM), Cairo, EGY

**Keywords:** autosomal-dominant polycystic kidney disease, frozen embryo transfer, intracytoplasmic sperm injection (icsi), preimplantation genetic testing for aneuploidy (pgt-a), preimplantation genetic testing for monogenic disorders (pgt-m)

## Abstract

Autosomal dominant polycystic kidney disease (ADPKD) is the most prevalent genetic kidney disorder, characterized by the development of cysts within the kidneys. ADPKD is primarily caused by mutations in the polycystic kidney disease genes 1 or 2 (PKD1 or PKD2), and less commonly by variants in other genes such as glucosidase II alpha subunit (GANAB), which have been associated with milder renal phenotypes and concurrent polycystic liver disease. Therefore, preimplantation genetic testing for monogenic disorders (PGT-M) offers a promising strategy for preventing the transmission of ADPKD-related mutations to offspring. When combined with preimplantation genetic testing for aneuploidy (PGT-A), this approach enhances the selection of genetically unaffected and chromosomally normal embryos, thereby improving implantation and live birth outcomes in affected couples. Here, we present a 28-year-old woman with ADPKD. In 2016, she delivered a male infant by cesarean section who was diagnosed with a more severe, early-onset form of PKD and sadly died at one year of age due to disease-related complications. In 2019, her renal function declined, necessitating a kidney transplant and subsequent immunosuppression. Three years later, after intrauterine device (IUD) removal, she experienced 1.5 years of secondary infertility before seeking fertility treatment. In 2022, she underwent an antagonist protocol after consulting a nephrology staff member. Subsequently, hormone replacement therapy was initiated for frozen embryo transfer, with endometrial preparation completed by day 20 of her cycle. Genetic counseling and a detailed pedigree-based family history were obtained. Genetic testing using targeted next-generation sequencing (NGS) was performed and revealed a novel in-frame deletion-insertion variant in the PKD2 gene (NM_000297.4:c.278_284delinsAGGAGGAGGTTATCCTCCTCCTCCCCGC) in a heterozygous state. The variant was classified as a variant of uncertain significance (VUS) according to the American College of Medical Genetics and Genomics (ACMG) and the Association for Molecular Pathology (AMP) guidelines, and is associated with ADPKD with or without polycystic liver disease. Database (ClinVar, gnomAD, Varsome) comparison confirmed its novelty and absence in population datasets. Despite uncertain pathogenicity, the strong genotype-phenotype correlation with classical ADPKD manifestations and the patient’s kidney transplant support its potential clinical relevance. The patient underwent combined PGT-M and PGT-A to exclude embryos carrying the PKD2 variant. Among the four embryos analyzed, one unaffected euploid female embryo was selected for transfer. Fourteen days later, a positive pregnancy test and subsequent ultrasound confirmed a single intrauterine gestation, culminating in a successful pregnancy and the delivery of a healthy newborn.

## Introduction

Autosomal dominant polycystic kidney disease (ADPKD) is the most common inherited kidney disorder, with prevalence estimates ranging from one in 400 to one in 1,000 in many populations, although regional and methodological variation exists [1,2]. Multiple extrarenal complications, including liver cysts, intracranial aneurysms, and cardiac valvular disease, as well as enlarged kidneys with expanding cysts and hypertension, demonstrate that ADPKD is a systemic disorder [3].

There is a clear genetic basis for ADPKD, a heterogeneous disorder resulting from gene mutations in the polycystic kidney disease 1 gene (PKD1) or polycystic kidney disease 2 gene (PKD2). To date, more than 1,500 mutations in the PKD1 and PKD2 genes have been detected in ADPKD patients. Mutations in the PKD1 gene account for approximately 78-85% of patients and cause more severe forms of nephropathy than mutations in the PKD2 gene do [3-5]. Furthermore, approximately 50% of the offspring of parents with ADPKD are at risk of inheriting the disease [6]. Protein-truncating variants that disrupt the protein-coding sequence in PKD1 or PKD2 account for a substantial portion of ADPKD cases and are reported to have 100% disease penetrance. Missense PKD1/PKD2 variants have also been linked to ADPKD, but with incomplete and variable penetrance [7]. Some unexplained cases may be due to variants in other genes such as glucosidase II alpha subunit (GANAB), DnaJ heat shock protein family (Hsp40) member B11 (DNAJB11), hepatocyte nuclear factor 1 beta (HNF1B), and alpha-1,2-mannosyltransferase ALG9 (ALG9) [8,9].

The clinical characteristics of ADPKD include the development of cysts in one or both kidneys; the number of these cysts increases over time, leading to nephromegaly and chronic renal failure. The extrarenal manifestations of the disease include cysts in other organs, such as the liver, seminal tract, and pancreas, as well as intracranial aneurysms. Moreover, men with ADPKD might present with infertility or subfertility caused by cysts in the seminal tract [4-6,10].

Most ADPKD patients have a positive family history of ADPKD and may pass the disease on to their children. Therefore, early genetic testing is necessary for early recognition and counseling the family on disease prognosis and management options [11]. However, approximately 10-15% of patients report no family history of the disease and harbor de novo mutations; these patients present mild phenotypes arising from PKD2 mutations and non-truncating PKD1 mutations or mosaicism. Many phenotypes are linked to the condition, ranging from newborns with enormous cystic kidneys to patients whose kidney function maintains adequate levels well into elderly age [12]. It has been recognized that some children develop more severe ADPKD than the typical course, and they may even experience renal failure (stage 5 chronic kidney disease) early in life. These children typically exhibit symptoms earlier in life, including oligohydramnios, pulmonary hypoplasia, and severe respiratory distress after birth, which can even mimic the much more severe autosomal recessive polycystic kidney disease (ARPKD) [13].

Several studies have highlighted the growing use of preimplantation genetic testing for monogenic disorders (PGT-M) since 2009, demonstrating its advantages for reducing the risk of disease transmission in couples affected by ADPKD [14-16]. PGT-M is performed as part of the in vitro fertilization (IVF) process, which offers a reproductive option for couples with disease-causing genetic variants, facilitating the identification and implementation of embryos without the disease, reducing disease transmission by >95% [17].

Chromosomal abnormalities, particularly aneuploidy, are well-known causes of implantation failure in IVF and early pregnancy loss. Consequently, employing preimplantation genetic testing for aneuploidy (PGT-A) is a promising option for decreasing the number of embryos transferred during IVF procedures and increasing the live birth rate per transfer [18].

Here, we describe a case of successful prevention of ADPKD transmission from a woman diagnosed with the disease who underwent a kidney transplant. Using a combined PGT-M and PGT-A strategy, she conceived a fetus via intracytoplasmic sperm injection (ICSI).

This study was previously published as a preprint on Research Square on December 8, 2024.

## Case presentation

A 28-year-old woman diagnosed with polycystic kidney disease (PKD) experienced preeclampsia during her previous pregnancy in 2016. She gave birth to a male fetus through cesarean section (CS), and the infant was also diagnosed with PKD, but with more severe and earlier age of onset symptoms. Unfortunately, the infant passed away a year later due to complications related to the disease. In 2019, the kidney function of the patient deteriorated, and she underwent a kidney transplant followed by immunosuppression. Three years later, the patient had an intrauterine device (IUD) removed and experienced 1.5 years of secondary infertility before seeking reproductive assistance. Given the previous obstetric history of the patient, a combination of PGT-M and PGT-A was recommended to exclude embryos carrying PKD-causing genetic variants. In December 2022, the patient underwent an antagonist protocol after consulting with the nephrology staff. Five oocytes were obtained through transvaginal ultrasound-guided oocyte retrieval. Of these oocytes, four were injected for ICSI, and the four resulting embryos were then biopsied for PGT-M and PGT-A analysis.

She had no history of smoking or alcohol consumption, and exposure to mutagens was unknown. Genetic counseling was performed, and a detailed family history was obtained. Following counseling, genetic testing was conducted using next-generation sequencing (NGS). All genes included in the test were evaluated for single-nucleotide variants (SNVs), small insertions and deletions (indels), as well as large deletions and duplications (del/dup) affecting copy numbers. Data analysis and interpretation were performed using the NGS pipeline version 1.1 (San Diego, CA: Vallard and Melendrez, SourceForge). Raw BCL files were converted to FASTQ files using bcl2fastq (Illumina) (San Diego, CA: Illumina, Inc.), and sequences were aligned to the human reference genome using the Burrows-Wheeler Aligner (BWA) program (San Diego, CA: Heng Li, SourceForge). The variants were interpreted according to the American College of Medical Genetics and Genomics (ACMG) guidelines, and patient phenotypes were classified as pathogenic (P), likely pathogenic (LP), unknown significance (VUS), likely benign (LB), or benign (B).

A novel in-frame deletion-insertion was identified in the PKD2 gene, described as NM_000297.4:c.278_284delinsAGGAGGAGGTTATCCTCCTCCTCCCCGC, corresponding at the genomic level to NC_000004.12:g.88008011_88008017delinsAGGAGGAGGTTATCCTCCTCCTCCCCGC (GRCh38). This alteration results in a change in the protein NP_000288.1:p.Gly93_Glu95delinsGluGluGluValIleLeuLeuLeuProAla, replacing three amino acids (Gly-Glu-95) with 10 residues near the N-terminal region of polycystin-2. Consequently, the variant was classified as a heterozygous variant of uncertain significance (VUS) according to ACMG guidelines.

The variant was analyzed across multiple public databases, including ClinVar (https://www.ncbi.nlm.nih.gov/clinvar/), gnomAD (https://gnomad.broadinstitute.org/), VarSome (https://varsome.com/), and Ensembl (https://www.ensembl.org/). No matching record or rsID was found, confirming its novel status. The variant corresponds to the genomic change NC_000004.12:g.88008011_88008017delinsAGGAGGAGGTTATCCTCCTCCTCCCCGC (GRCh38), transcript NM_000297.4:c.278_284delinsAGGAGGAGGTTATCCTCCTCCTCCCCGC, and protein NP_000288.1:p.Gly93_Glu95delinsGluGluGluValIleLeuLeuLeuProAla. This alteration was detected in the heterozygous state.

Defects in the PKD2 gene cause PKD with or without polycystic liver disease [MIM:613095]. Given the absence of this variant in population databases and its alignment with the patient’s phenotype, it is considered a VUS pending additional functional or segregation data.

**Figure 1:**
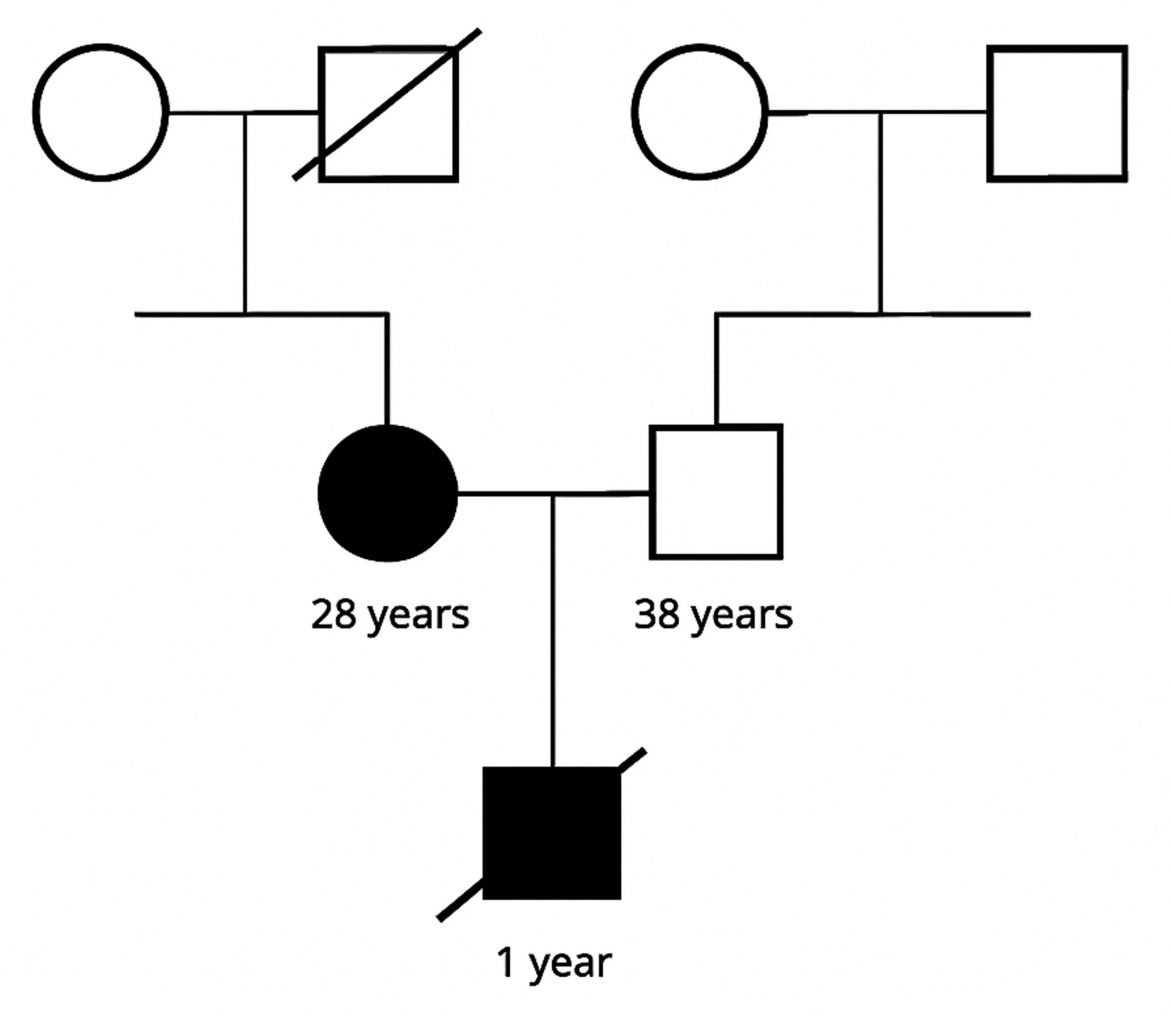
Genetic counseling and a detailed family history and pedigree obtained during the first visit.

The patient had no family history of ADPKD, suggesting that this mutation arose de novo. Unfortunately, her father had passed away before the counseling session, and her mother, who is apparently healthy, underwent linkage analysis that confirmed the absence of the variant in her. The pedigree was obtained during the first visit (Figure 1).

Genetic testing for the proband was performed on a peripheral blood sample, confirming that the variant is constitutional (germline) rather than somatic. This interpretation is further supported by the family history, i.e., the couple’s first child was affected by PKD and died at one year of age, exhibiting a more severe phenotype than the mother. This observation confirms the heritability of the variant and excludes postzygotic mosaicism as the underlying mechanism.

We recognize that the identified variant remains categorized as a VUS under ACMG/AMP guidelines. Nevertheless, the proband’s clinical presentation of progressive cystic kidney disease requiring transplantation in adulthood and the recurrence of disease in the offspring provide strong genotype-phenotype correlation supporting its clinical relevance.

**Figure 2:**
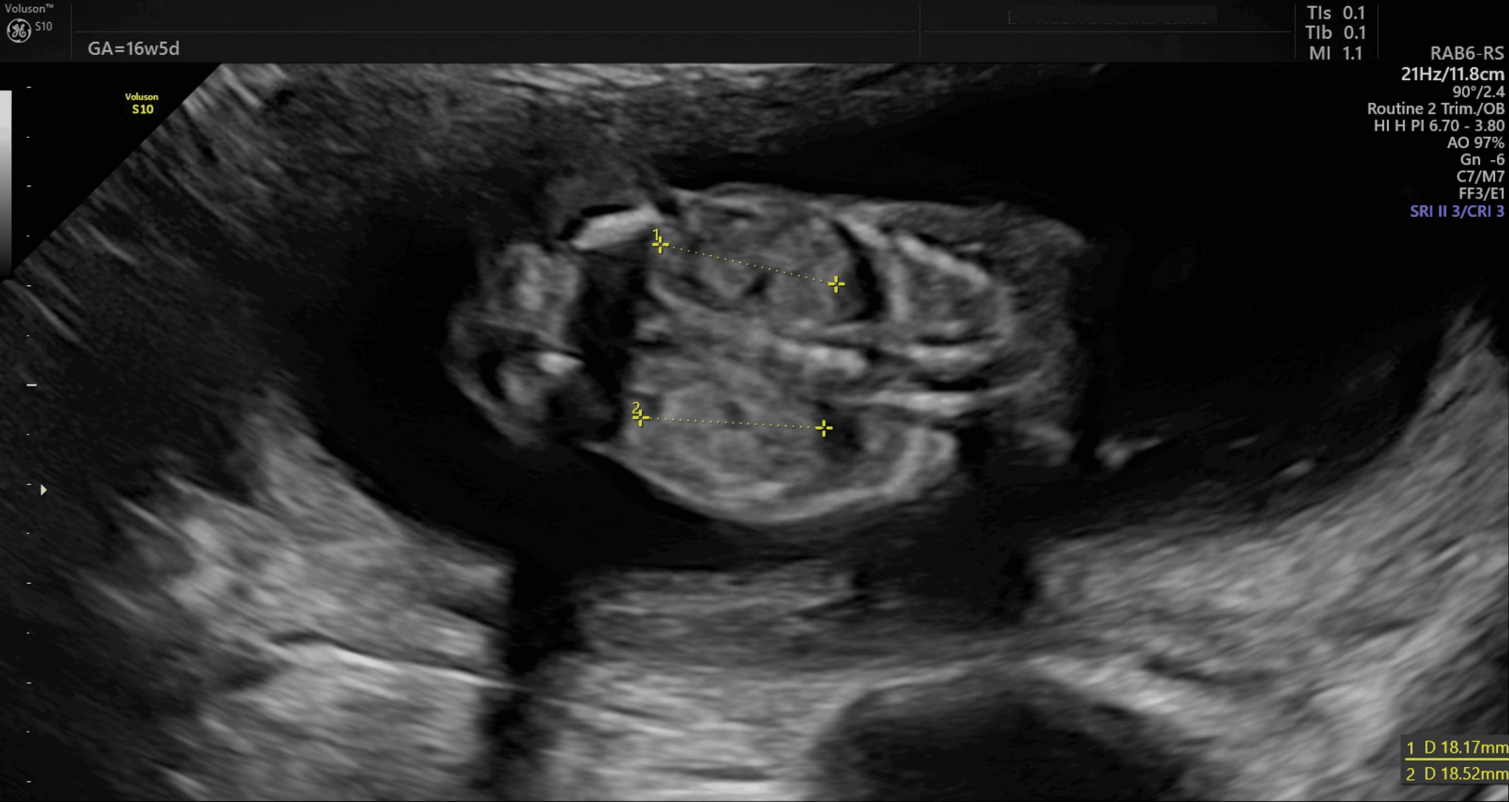
Ultrasound image showing the normal shape of both fetal kidneys.

**Figure 3:**
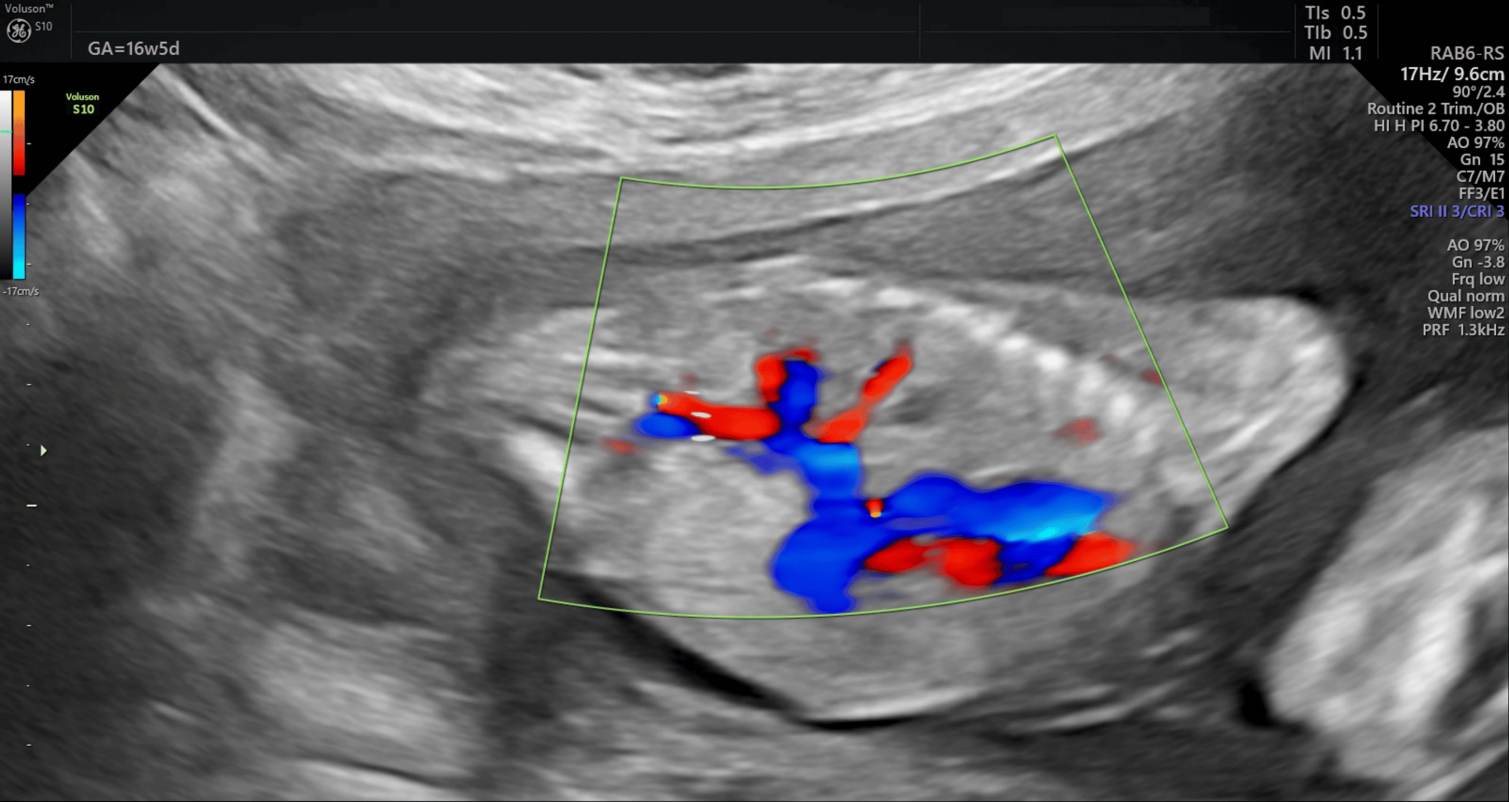
Doppler ultrasound image showing the normal blood supply of both fetal kidneys.

The test results revealed one healthy female embryo and three affected embryos. The patient subsequently began the hormone replacement therapy protocol for frozen embryo transfer, and her endometrium was ready for blastocyst transfer on day 20 of her cycle. A pregnancy blood test was performed 14 days after the transfer, and the result was positive; a subsequent ultrasound revealed a single gestational sac, confirming pregnancy. Prenatal ultrasound during pregnancy revealed normal fetal development (Figures 2, 3).

Additionally, the patient had normal kidney function and blood pressure. At 29 weeks of gestation, ultrasound revealed polyhydramnios, with the deepest vertical pocket measuring 10 cm. At 33 weeks of gestation, premature rupture of membranes and infrequent uterine contractions occurred. An urgent lower segment CS was subsequently performed, and a single living female fetus weighing 2,200 g was delivered. The baby was admitted to the neonatal intensive care unit (NICU) due to respiratory distress and received supplemental oxygen therapy, initially through continuous positive airway pressure (CPAP) for one day, followed by nasal oxygen for another day. A specialized nephrologist assessed the baby, and the findings of her renal and general examinations were normal. Therefore, the infant was discharged; throughout follow-up, she achieved normal developmental milestones. Appropriate written informed consent for publication and personal medical information of this case report was requested and obtained.

## Discussion

ADPKD is a genetic disorder characterized by the development of renal cysts, progressive impairment of kidney function, and chronic renal failure. PGT-M is an effective method for the early detection and prevention of genetic diseases being transmitted from parents to children. In our study, we describe the successful implementation of combined PGT-M and PGT-A to select embryos for a woman carrying a VUS variant in the PKD2 gene causing ADPKD, who underwent ICSI and conceived a healthy fetus. This PKD2 variant (NM_000297.4:c.278_284delinsAGGAGGAGGTTATCCTCCTCCTCCCCGC) is not listed in ClinVar, gnomAD, or dbSNP, and no rsID has been assigned, indicating that it is a novel variant. This study represents the first documented successful application of PGT among patients with ADPKD in the Middle East. Moreover, we are reporting a novel variant in the studied family.

In a 25-year cohort study conducted in the Netherlands by Snoek et al., 53% of couples with monogenic kidney disease chose to undergo PGT [14]. The study indicated that mothers were affected by kidney diseases more often than fathers were, with 38% of the cases involving ADPKD. Among the 537 embryos that were biopsied, 35% were found to be genetically unaffected and suitable for transfer. Additionally, approximately two-thirds of these couples experienced one or more live births of unaffected fetuses, which is comparable to the outcomes of IVF [18]. The fact that the mother is the proband corresponds with our study, which may be explained by their findings that mothers are more likely than fathers to be affected. In line with the anticipated success rate of PGT-M in APKD, the IVF outcome in our study was successful.

The majority of patients (93%) who present with ADPKD had mutations in the PKD1 and PKD2 genes, which encode polycystin 1 and 2, respectively. PKD1 mutations account for 85% of ADPKD, while PKD2 mutations account for approximately 15% of cases [19]. Several reports in the literature have highlighted the promising PGT findings among patients with monogenic kidney disease. Chaperon and his colleagues have indicated that ADPKD was the primary kidney disease targeted for PGT-M at their reference laboratory over the past decade, highlighting the utility of genetic testing in reducing the risk of disease transmission with a significantly low probability of misdiagnosis (<1%). It was shown that about 80% of Chinese patients with ADPKD, who desire children, were interested in PGT. About half of those who were unsure about PGT were concerned about the technical safety [20,21].

In a 10-year cohort study in China, the cumulative pregnancy/live birth rate was 54.69% among couples at risk for genetic kidney disease, primarily PKD (34.3%). Within their cohort, a total of 344 embryos were assessed; 20.6% of these embryos were unaffected euploid embryos, and 150 embryos (43.6%) were free of kidney disease-related mutations and deemed suitable for transfer. Additionally, it was indicated that 80% of couples at risk for inheriting a monogenic disease in their children favor PGT-M over other options, including adoption, donor gamete use, or prenatal diagnosis [22]. These findings support our result by showing that embryo screening in genetic kidney disease yields substantial live birth rates. Our case similarly obtained an unaffected embryo and a successful pregnancy, reinforcing the feasibility of PGT in ADPKD. Differences in mutation type, patient selection, and clinical context (e.g., post-transplant status) mean we cannot directly equate rates, but the concordance provides supportive external validation. The broad application of these techniques in various countries, including the Middle East and East Asia, such as South Korea, remains limited.

Berckmoes et al. conducted a study to investigate the factors influencing the success rate of couples undergoing PGT for PKD. The live birth delivery rates were 37.7% for fresh embryo transfers and 39.4% for frozen embryo transfers. Additionally, the observed cumulative live birth rate was 57.8%, compared with an expected cumulative live birth rate of 77.4%. Moreover, the authors concluded that only maternal age, a well-established risk factor for adverse pregnancy outcomes in general, was significantly associated with the live birth delivery rate [23]. Pardo et al. investigated the effects of combining PGT-M and PGT-A on the number of aneuploid embryos and the number of cycles yielding transferable embryos in patients with ADPKD. Among the 289 embryos analyzed, 49.3% were transferable. Additionally, 94.0% of the cycles resulted in transferable embryos, with 94.0% of the cycles involving PGT-M and 69.9% involving the combined PGT approach [24]. Like the study by Berckmoes et al., the authors suggested that maternal age is the determining factor for aneuploidy rather than male infertility [23]. In the present study, the mother's age risk factor was low, and the embryo was transferred following PGT-M and PGT-A regardless of age to avoid chromosomal abnormalities. The IVF resulted in a healthy, unaffected female offspring, which is consistent with the studies’ anticipated live birth delivery rate.

Liu et al. conducted a retrospective study involving 352 Chinese couples with nephropathy-related diseases. Of these, the primary indication for referral for PGT-M was ADPKD (81.7%). Among the tested embryos, 76 were transferable and resulted in 38 live births, with 37 resulting from the transfer of non-pathogenic embryos [25]. However, the type of mutation in the PKD2 gene in our case differed from those reported in their cohort. Our study reported a novel VUS in-frame deletion-insertion variant in the heterozygote state in the PKD2 gene, with phenotype-genotype correlation strongly matching the patient’s medical condition.

Several studies from South Asia document the burden and genetic diversity of ADPKD in the region and underscore the relevance of reproductive counseling for affected families. In Korea, although there are no published studies on PGT for ADPKD, a successful application of PGT has been reported for a carrier of mucopolysaccharidosis type II and couples with Charcot-Marie-Tooth disease. Pandita et al. and Raj et al. had made cohort studies and described PKD1 and PKD2 variants mutations among the Asian-Indian population. They also described the clinical course and highlighted the need for local genetic service [26,27]. Kundu et al. and Luo et al. highlighted that secondary subfertility is on the rise in South Asia, which emphasizes the significance of easily accessible reproductive alternatives, such as PGT-M, for families at risk of passing on ADPKD. Furthermore, ADPKD is recognized as one of the approved indications for PGT in Korea, highlighting the potential of this technique for the early detection and prevention of disease transmission to children in Korea [28,29].

Here, the patient had a history of preeclampsia during her previous pregnancy and experienced polyhydramnios and premature rupture of membranes, followed by delivery during her most recent pregnancy. In a prior case-control study, women with ADPKD presented a non-significantly increased risk of spontaneous abortion and premature delivery. However, the risks of preeclampsia, hypertension, proteinuria, renal dysfunction, edema, and urinary tract infections were significantly different between the groups [23]. Therefore, close monitoring of pregnant women with ADPKD is necessary.

Genetic counseling for couples with ADPKD considering PGT-M should include a discussion about success rates, costs, waiting times, technical limitations, and maternal health risks associated with the procedure [14]. Despite successful reports of PGT for ADPKD, its use for ADPKD may raise several regulatory and ethical concerns. These issues include the absence of government regulation and inconsistent consent processes, the potential for technical errors, high costs, concerns about equity, and risks associated with pregnancy for mothers with kidney disease [24]. Recently, KDIGO and the European Society of Human Reproduction and Embryology (ESHRE) issued good-practice recommendations suggesting PGT for couples with confirmed ADPKD and accepting the discarding of unimplanted embryos affected by ADPKD [10,25].

Therefore, assessing risks and benefits and receiving genetic counseling from an experienced clinician is highly recommended. Additionally, future studies and the development of best-practice statements are needed to optimize the application of this technology in patients with ADPKD.

## Conclusions

In conclusion, although the identified variant in the PKD2 gene is classified as a VUS, according to the ACMG, the patient’s medical conditions are consistent with ADPKD. This strong genotype-phenotype correlation raises the possibility that this novel variant contributes to the disease in this patient. PGT-M is a promising strategy for decreasing the transmission of ADPKD from couples diagnosed with the disease to their future offspring. Therefore, multidisciplinary management, including genetic counseling, remains essential to guide variant interpretation, provide reproductive options such as PGT-M, and ensure long-term follow-up for potential variant reclassification.

Additional analysis of ADPKD patients undergoing PGT-M is necessary to evaluate the effects of PGT-M on both clinical practice and maternal and fetal outcomes. We highlight that additional functional research and segregation analysis are required to justify the variant's possible future classification.
